# Optimal empiric treatment for KPC-2-producing *Klebsiella pneumoniae* infections in critically ill patients with normal or decreased renal function using Monte Carlo simulation

**DOI:** 10.1186/s12879-021-06000-2

**Published:** 2021-03-26

**Authors:** Guoan Wang, Wei Yu, Yushan Cui, Qingyi Shi, Chen Huang, Yonghong Xiao

**Affiliations:** 1 Department of Respiratory Medicine, Ningbo Medical Center Lihuili Hospital, Ningbo, 315000 China; 2 State Key Laboratory for Diagnosis and Treatment of Infectious Disease, Collaborative Innovation Center for Diagnosis and Treatment of Infectious Diseases, The First Affiliated Hospital, College of Medicine, Zhejiang University, Hangzhou, 310003 China

**Keywords:** Population pharmacokinetics/pharmacodynamics model, Renal function, Tigecycline, Colistin methanesulfonate, Fosfomycin, KPC-2-producing *Klebsiella pneumoniae*

## Abstract

**Background:**

Limited clinical studies describe the pharmacodynamics of fosfomycin (FOS), tigecycline (TGC) and colistin methanesulfonate (CMS) in combination against KPC-producing *Klebsiella pneumoniae* (KPC-Kp). Population pharmacokinetic models were used in our study. Monte Carlo simulation was conducted to calculate probability of target attainment (PTA) and cumulative fraction of response (CFR) of each agent alone and in combination against KPC-Kp in patients with normal or decreased renal function.

**Results:**

The simulated regimen of FOS 6 g q8h reached ≥90% PTA against a MIC of 64 mg/L in patients with normal renal function. For patients with renal impairment, FOS 4 g q8h could provide sufficient antimicrobial coverage against a MIC of 128 mg/L. And increasing the daily dose could result to the cut-off value to 256 mg/L in decreased renal function. For TGC, conventional dosing regimens failed to reach 90% PTA against a MIC of 2 mg/L. Higher loading and daily doses (TGC 200/400 mg loading doses followed by 100 mg q12h/200 mg q24h) were needed. For CMS, none achieved 90% PTA against a MIC of 2 mg/L in normal renal function. Against KPC-Kp, the regimens of 200/400 mg loading dose followed by 100 q12h /200 mg q24h achieved > 80% CFRs regardless of renal function, followed by CMS 9 million IU loading dose followed by 4.5/3 million IU q12h in combination with FOS 8 g q8h (CFR 75–91%).

**Conclusions:**

The use of a loading dose and high daily dose of TGC and CMS in combination with FOS can provide sufficient antimicrobial coverage against critically ill patients infected with KPC-Kp.

**Supplementary Information:**

The online version contains supplementary material available at 10.1186/s12879-021-06000-2.

## Introduction

*Klebsiella pneumoniae* is an increasingly important bacterial pathogen that causes severe lift-threatening diseases [1]. However, data from China Antimicrobial Surveillance Network (CHINET) indicated the resistance rate to imipenem in *K. pneumoniae* isolates has increased from 0.4% in 2005 to 25.0% in 2018 [2, 3]. The increasing emergence of carbapenem-resistant *K. pneumoniae* (CRKP), especially *Klebsiella pneumoniae* carbapenemase (KPC)-producing *K. pneumoniae* (KPC-Kp), has become an urgent public health problem in healthcare settings, resulting in higher morbidity, mortality and medical cost [4, 5]. The risk of high mortality related to these infections was inappropriate empirical antimicrobial treatment [6]. However, the paucity of new classes of antibiotics with which to treat such circumstance has led to regain significant interest in the revival of fosfomycin (FOS), tigecycline (TGC), and colistin methanesulfonate (CMS) as last-resort drugs [7]. Therefore, evaluation of the efficacy of these alternative options is necessary to manage the immediate threat of CRKP in the the ‘bad bugs, no drugs’ era, in addition to facilitating the development and clinical authorization of novel antimicrobials [8]. As an in vitro susceptibility is insufficient to choose rational antibiotic or dosing regimens in clinic, the introduction of population pharmacokinetics (PK) with monte carlo simulation (MCS) integrates population-PK parameters and population-minimum inhibitory concentration (MIC) pathogen data together to calculate the likelihood of achieving a certain target [9]. This approach may be applied to optimize dosing regimens, maximize the desired effects, and re-evaluate reasonable clinical breakpoints.

Colistin (CST) and TGC show favourable in vitro activity against CRKP [10]. However, the role of CST and TGC in the treatment of severe nosocomial infections remains controversial. The reason may be a great inter-individual variability in the population PK and heteroresistance for CMS. And for TGC, a large volume of distribution and low concentrations in blood, urine, and epithelial lining fluid of the lungs were observed [11–13]. Some experts have revealed the current recommended dosage of TGC and CMS may be suboptimal, and higher doses should be considered [14, 15]. Furthermore, several studies have shown that combination therapy resulted to the promising outcome than monotherapy in combating multidrug-resistant infections, and the dosing regimens included TGC or CMS were associated with lower mortality [16]. And for FOS, it may remain active against a considerable proportion of CRKP, especially for carbapenem-resistant *Enterobacteriaceae* [10]. It can be used in the management of difficult-to-treat infections combined with other antimicrobial agents [17]. There is confusion regard to whether FOS displays time- or concentration-dependent bactericidal activity [18, 19]. It seems that this depends on the microorganism under study. Therefore, two different estimations of PK/PD indices for FOS may be done in our analysis. Although several studies regarding the MCS of FOS, TGC and CMS have been done, most of these evaluations were evaluated primarily in a monotherapy setting, and combination antimicrobial synergy studies using this method are scarce.

To date, there have been limited studies concerning on the optimal dosage regimens of the three antibiotics for the treatment of KPC-Kp infections in our region. The aim of our study was to: (i) re-evaluate reasonable clinical breakpoints of FOS, TGC and CST using MCS; (ii) assess the efficacy of three candidate antibiotics against KPC-Kp by mono- or combination therapy; (iii) find the prompt initiation of appropriate antimicrobial therapy against KPC-Kp.

## Materials and methods

### Bacterial isolates

The MIC distributions were obtained from our previous study [20]. Briefly, a total of 136 clinical KPC-Kp were collected from different hospitals in China. Antimicrobial susceptibility testing for FOS was performed by the agar dilution method and the MICs of TGC and CST were tested by broth microdilution method according to Clinical and Laboratory Standards Institute (CLSI) guidelines [21]. Different combinations of antimicrobials were tested to estimate synergistic activity by the chequerboard test [20]. The MIC_90_ values for FOS, TGC and CST against KPC-Kp used in the present study were 1024 mg/L, 4 mg/L and 0.5 mg/L, respectively (Table S1). For the combined therapy, the majority of the MICs were lower than that in monotherapy, and the MIC_90_ were correspondingly decreased to 1/2–1/16.

### Simulation of FOS, TGC and CMS pharmacokinetics

The demographics of 10,000 virtual patients were first simulated in a 50/50 ratio of males and females. Height was assumed to be normally distributed, with the height of males being 1.71 ± 0.06 and females being 1.59 ± 0.06 in China [22]. And the distributions of body mass index (BMI) among Chinese elderly were 22.76 ± 3.2 and 22.97 ± 3.5 for males and females, respectively [23]. The relationship between height and weight was shown as the following equations [24]: WT_male/female_ = 2.2 × BMI_male/female_+ 3.5 × (HT_male/female_-1.5), where WT refers to weight and HT refers to height. The age of the population was uniformly distributed between 60 and 90. Serum creatinine (S_Cr_) in critically ill patients with normal renal function were 0.7 ± 0.05 and 0.6 ± 0.05 mg/dl for males and females, respectively, whereas 1.5 ± 0.15 and 1.2 ± 0.15 mg/dl for S_CR_ with renal decreased function [25]. The modification of renal disease (MDRD) equation was introduced to calculate Creatinine clearance (CrCL): CrCL = 186 × S_Cr_^1.154^ × age^-0.203^ and CrCL =186 × S_Cr_^1.154^ × age^-0.203^ × 0.742 for males and females, respectively [26].

The population PK final model for FOS in critically ill patients with CrCL ranged from 30 to 300 mL/min was a two-compartment linar model. The parameters of clearance (CL), volume of central compartment (V_C_), intercompartmental clearance (Q), and volume of peripheral compartment (V_P_) were derived from Parker et al. [27]. In their model, CrCL and WT were influential covariates related to CL and V_C_, respectively. The equations for the population CL and V_C_: CL = 5.57 × (CrCL/90), and V_C_ = 26.5 × (WT/70)^0.75^. V_P_ and Q were 22.3 l and 19.8 l/h, respectively. The between-subject variability in CL and Vc were 91.9 and 39%, respectively. FOS has negligible plasma protein binding [28].

The population PK model for TGC was derived from patients infected with intra-abdominal infections or complicated skin and skin- structure infections [29]. A two-compartment model was used to depict the time-concentration curve for TGC. The covariate relationship was associated with CrCL, WT and sex: CL = 15.7 × (CrCL/88.3)^0.25^ + 0.093 × (WT - 80) + 3.23 × (1-sex), where sex is an indicator variable with a value of 1 for females and 0 for males. The between-subject variability in CL and Vc were 36.2 and 43.7%, respectively. Of note, previous studies have shown that differences in CrCL were not expected to substantially affect TGC exposure [30]. The population PK model derived from Wart et al. was predicted to have slightly higher AUC values in modern renal impairment comparted to normal renal function [29]. This increase in TGC exposure was not expected to adjust doses for patients with moderate renal impairment.

The population PK model for CMS in critically ill patients was described by a linear model comprising two-compartment [31]. The total CMS clearance was modeled as a function of CrCL and two random effects, CLR_SLOPE_ and CLNR_CMS_. The equation for the population CL: CLT_CMS_ = CrCL×CLR_SLOPE_ + CLNR_CMS_, where CLT_CMS_ refers to the total intrinsic clearance for CMS, CLR_SLOPE_ refers to the slope of the relationship between renal clearance of CMS and creatinine clearance and CLNR_CMS_ refers to non-renal clearance of CMS. The between-subject variability in CLR_SLOPE_ and CLNR_CMS_ were 70 and 36%, respectively.

### Pharmacokinetics/pharmacodynamics target (PK/PD)

FOS displays time- or concentration-dependent bactericidal effects depend on the type of Gram-negative isolates, and %T > MIC and AUC_24_/MIC is the PD index most closely linked to the efficacy. As PK/PD targets, we selected %T > MIC > 70% for all pathogens, and AUC_24_/MIC ≥24 for net stasis of *Enterobacteriaceae*, based on the study by Lepak et al. [32]. And from previous studies, concentration-dependent killing was demonstrated against *Enterococcus faecium*, *E. coli*, and *P. mirabilis* [33, 34]. Thus, we chose AUC_24_/MIC as the main PK/PD target. For %T > MIC, equations were used to calculate concentrations by using a two-compartment model for FOS [35]. For TGC and CMS, the antibacterial activity was found to correlate with the PK/PD index calculated by AUC_24_/MIC. The values of ≥6.93 and ≥ 60 were necessary for TGC and CMS, respectively, based on the previous studies [31, 36]. These PK/PD targets, described above, were either used alone or in combination.

### Monte Carlo simulation

A 10,000 patient MCS was conducted to calculate the probability of target attainment (PTA) and cumulative fraction of response (CFR) of each dosage regimen against bacterial population using Crystal Ball software (version 11.1.2.4; Oracle) to evaluate their efficacy. The following dosage regimens were evaluated: FOS 4 g/6 g/8 g every 8 h (q8h) as 0.5-h and 4-h infusions and FOS 16 g continuous infusion, TGC 100/200 mg loading dose followed by 50/100 mg every 12 h (q12h), TGC 200/400 mg loading dose followed by 100/200 mg every 24 h (q24h), CMS 2/3/4.5 million IU q12h and CMS 9 million IU loading dose followed by 3/4.5 million IU q12h. The PTA value of each drug regimen was considered to be adequate when a target of ≥0.9 was reached. The CFR was calculated as the proportion of %PTA of each MIC according to the MIC distributions. An optimal regimen was defined as achieving ≥90% CFR against a population of organisms whereas a CFR between 80 and 90% was associated with moderate probabilities of success [37, 38].

## Results

### %PTA with different dosing regimens

**Fig. 1:**
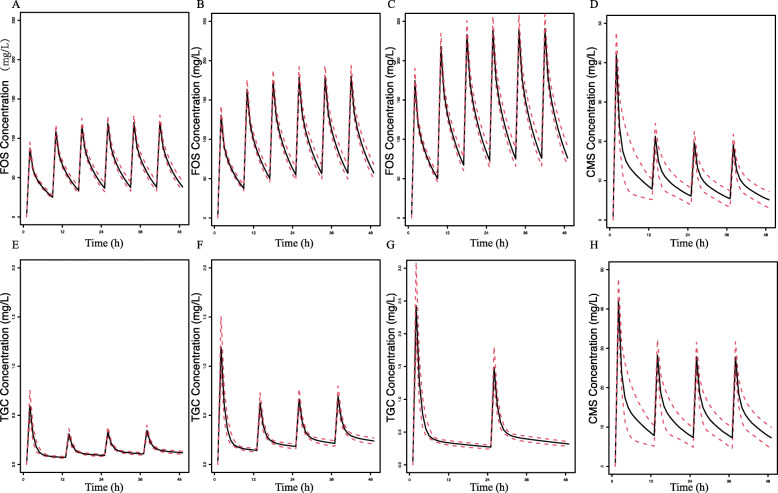
Simulated median and 95% prediction interval of FOS, CMS and TGC concentrations in serum in male patients with normal renal function: **a** FOS 4 g q8h; **b** FOS 6 g q8h; **c** FOS 8 g q8h; **d** CMS 9 MIU loading. Dose followed by 3 million IU q12h; **e** TGC 100 mg loading dose followed by 50 mg q12h; **f** 100 mg loading dose followed by 50 mg q12h; **g** 400 mg loading dose followed by 200 mg q24h; **h** 9 million IU loading dose followed by 4.5 million IU q12h. Black soid line, simulated median concentrations of drugs; red dotted line, 95% prediction interval concentrations of drugs. FOS, fosfomycin; CMS, colistin methanesulfonate; TGC, tigecycline

**Fig. 2:**
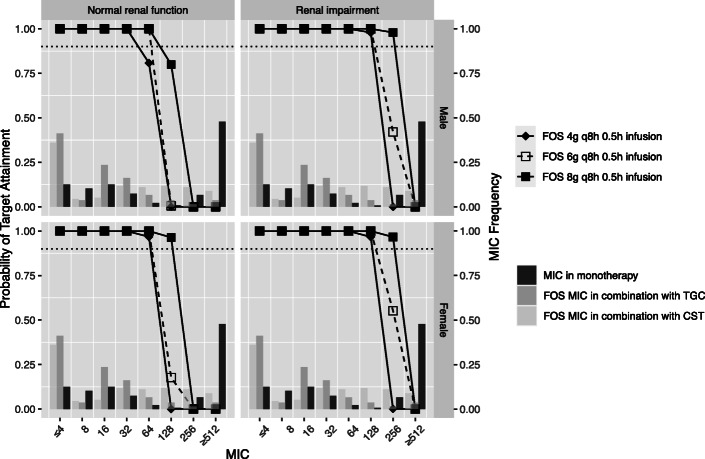
The MIC distribution of FOS in monotherapy or combination with TGC or CMS against 136 KPC-producing. *Klebsiella pneumoniae,* and probability of target attainment (PTA) of 24 AUC24/MIC for FOS dosing regimens in critically. Ill elderly patients with normal renal function (Left) and renal impairment (right). The dotted line indicates the PTA of 0.9. FOS, fosfomycin; TGC, tigecycline; CST, colistin

**Fig. 3:**
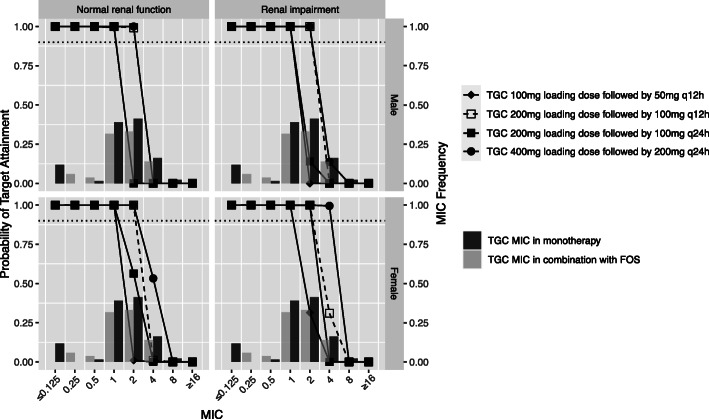
The MIC distribution of TGC in monotherapy or combination with FOS against 136 KPC-producing *Klebsiella. pneumoniae,* and probability of target attainment (PTA) of 6.93 AUC24/MIC for TGC dosing regimens in critically ill elderly patients with normal renal function (Left) and renal impairment (right). The dotted line indicates the PTA of 0.9. FOS, fosfomycin; TGC, tigecycline

**Fig. 4:**
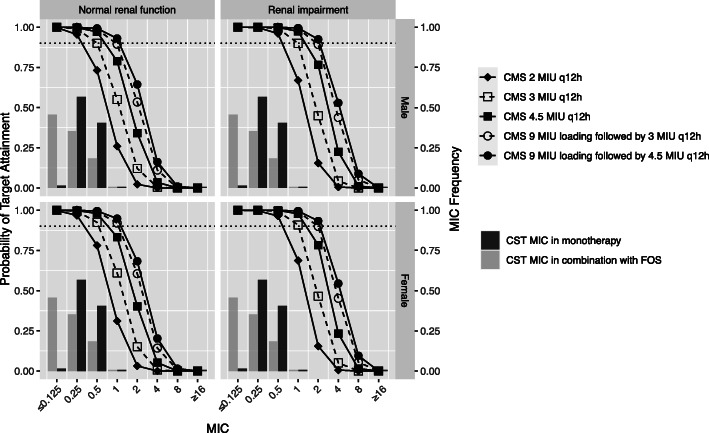
The MIC distribution of CMS in monotherapy or combination with FOS against 136 KPC-producing *Klebsiella pneumoniae,* and probability of target attainment (PTA) of 60 AUC24/MIC for TGC dosing regimens in critically ill elderly patients with normal renal function (Left) and renal impairment (right). The dotted line indicates the PTA of 0.9. FOS, fosfomycin; CST, colistin; CMS, colistin methanesulfonate; MIU, million IU

In this study, a bayesian-based dosing for FOS, TGC and CST in mono- or combination therapy was conducted to calculate PTA or CFR. The distribution of CrCL for male and female patients was shown in Figure S1. For the simulated normal renal function and renal impairment, the range of CrCL was 80 to 150 ml/min and 30 to 80 ml/min, respectively. Figure 1 showed the simulated median and 95% prediction interval of FOS, CMS, and TGC in male patients with normal renal function. The relationships between MIC and PTA for various dosing regimens and CrCL were presented in Figs. 2, 3 and 4. Based on the PK/PD target of AUC_24_/MIC > 24, FOS 6 g q8h reached ≥90% PTA at the susceptibility CLSI breakpoint for *Enterobacteriaceae* (MIC = 64 mg/L) in patients with normal renal function. And the cut-off for achieving ≥90% PTA was raised to 128 mg/L for FOS 8 g q8h in female normal renal function group (Fig. 2). For patients with renal impairment, FOS 4 g q8h could reach ≥90% PTA at a MIC of 128 mg/L. Increasing the daily dose (24 g/day) could result to the cut-off value to 256 mg/L. However, based on %T > MIC, only the simulated regimens of FOS 8 g q8h as a 0.5/4-h infusions reached ≥90% PTA against isolates with a MIC of 64 mg/L in the normal renal function (Figure S2). Similar results were also found in patients with renal impairment for FOS 16 g continuous infusion or FOS 6 g/8 g q8h as a 0.5/4-h infusion. Unfortunately, none of the FOS dosing regimens achieved ≥90% PTA against a MIC of 128 mg/L, regardless of renal function.

The PTAs were almost 100% against isolates with MIC ≤1 mg/L for all the simulated TGC regimens (Fig. 3). Increasing the daily doses could improve the cut-off MIC of susceptibility. Our results revealed that AUC/MIC for TGC 200 mg loading dose followed by 100 mg q12h reached 6.93 against isolated with MIC ≤2 mg/L as well as TGC 400 mg loading dose followed by 200 mg q24h regardless of renal function. Furthermore, TGC 200 mg loading dose followed by 100 mg q24h and 400 mg loading followed by 200 mg q24h in the female impaired renal function cohort also reached ≥90% PTA against a MIC of 2 mg/L and 4 mg/L, respectively.

For CMS, all the CMS dosing regimens, except for CMS 2 million IU q12h, achieved PTA target against the MIC_90_ of KPC-Kp regardless of renal function. The target attainment rates for simulated CMS regimens in normal renal function and decreased renal function (2 million IU q12h, 3 million IU q12h, 4.5 million IU q12h, 9 million IU loading dose followed by 3 million IU q12h, and 9 million IU loading dose followed by 4.5 million IU q12h) against isolates with MICs ≤0.25/0.5, ≤0.5/1, ≤0.5/1, ≤1/2 and ≤ 1/2 mg/L, respectively, exceeded 90% (Fig. 4). Notably, 90% PTA was achieved only in renal impairment treated with the two loading dose regimens at the susceptibility breakpoint (MIC ≤2 mg/L) from CLSI.

### %CFR of monotherapy or combination therapy

Table 1 summarized the CFRs for each dosing regimen of FOS in combination with TGC and CMS against the tested KPC-Kp. Our findings revealed that the CFRs were low (≤60%) in TGC 100 mg loading dose followed by 50 mg q12h in combination therapy, regardless of renal function or the dosage regimens of FOS. The PK/PD targets of ≥80% CFRs were achieved in FOS 8 g q8h in combination with TGC 200 mg loading dose followed by 100 mg q12h or 400 mg loading dose followed by 200 mg q24h in patients with normal renal function (Table 1). Of note, the simulated combination regimen of FOS 4 g q8h and TGC 400 mg loading dose followed by 200 mg q24h also achieved a promising CFR in female normal renal function population. Moreover, the values for FOS in combination with TGC were higher in patients with renal impairment. FOS 4 g q8h in combination with TGC 200 mg loading dose followed by 100 mg q12h reached ≥80% CFRs against KPC-Kp.

**Table 1:**
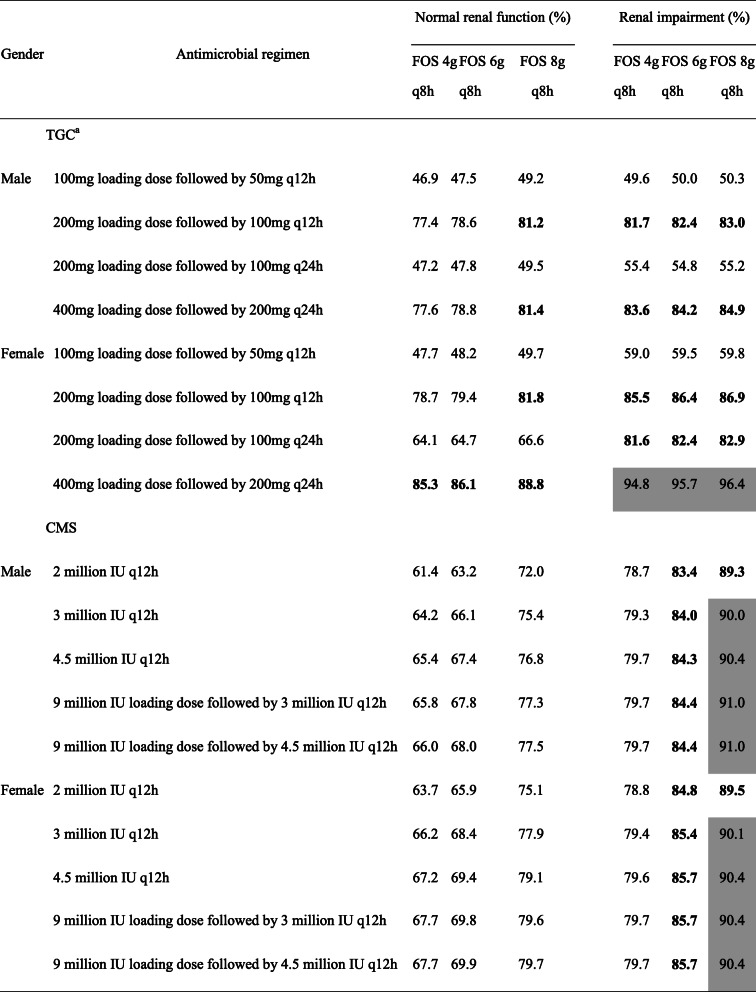
Cumulative fraction of response to TGC and CMS in combination with FOS against KPC-2-producing *K. pneumoniae*^a^

Gray shading indicates ≥90% CFR, and boldface indicates 80 to 90%

*FOS* fosfomycin, *TGC* tigecycline, *CMS* colistin methanesulfonate

^a^Pharmacodynamic target: AUC24/MIC ≥24 for FOS, AUC24/MIC ≥6.93 for TGC and AUC24/MIC ≥60 for CMS

For the combination regimens of FOS and CMS, none of the simulated FOS-CMS combinations achieved 80% CFR in patients with normal renal function, and the highest dose combination consisting of FOS 8 g q8h and CMS 9 million IU loading dose followed by 4.5 million IU q12h only resulted in approximately 80% CFR (Table 1). Due to the two loading dosing regimens of CMS are recommended in treating patients with CrCL > 60 mL/min and 30–60 mL/min, respectively, both of them showed promising response with above 85 and 90% CFR values in combination therapy with FOS 6 g q8h and 8 g q8h.

## Discussion

KPC-Kp are increasingly prevalent and has been becoming a global public health concern. This dilemma often resulted in early inappropriate antimicrobial therapy associated with a high-risk factor for the mortality rates [6]. The KPC-Kp, used in our study, were highly susceptible to CST, nevertheless showed limited susceptible to TGC and FOS. The reason may be the widely use of TGC in the treatment of Carbapenem-resistant Gram-negative bacterial infections in China. In this regard, it is critical to know local trends in resistance and population-MIC distributions in order to achieve better empirically therapeutic outcomes [39]. The adequate empirical antibiotic treatment should be considered local and recent data on antimicrobial resistance as well as inter-individual variation of PK behavior in virtual patients. Bayesian-based dosing for patients was conducted in our study to provide individualised dosing regimens from a patient’s own PK parameter estimates. Thus, a truly optimized regimen could be derived for each patient. This can improve the clinical cure rate, especially in critically ill patients infected with highly resistant pathogens, rather than the manufacturer’s prescribing information. To the best of our knowledge, this is the first and largest study to estimate the combined treatment of FOS with TGC or CMS against KPC-Kp using population-PK model in China. Our findings highlighted the importance of high dose TGC or CMS in combination with FOS against KPC-Kp. This would be useful in empirically treating patients infected with KPC-Kp or high risk factors of CRKP, as quite a few tertiary and secondary health care settings failed to afford the MIC results of FOS and CST in clinic.

The patients with CrCL of < 30 ml/min was not simulated in our study as such patients in the stage of end stage renal disease (ESRD) often require dialysis therapies. Several changes of antibiotics in absorption, distribution and metabolism would be noted after dialysis [40]. This depends on the characteristics of dialyzing membrane and drug, the rate of blood flow as well as the duration of therapy [40]. The simulated CrCL of > 30 ml/min was in accordance with the reported CrCL in critically ill patients [41].

FOS is being used frequently against multidrug-resistant organisms. Our data revealed that none of the FOS regimens in monotherapy was able to achieve PK/PD targets related to antimicrobial efficacy for KPC-Kp. Consistent with another PK study of FOS 8 g q8h in critically ill patients, a mean of C_max_ 307 mg/L also failed to reach the target because of the high MICs [42]. Fortunately, the combination with TGC brought the FOS MIC_90_ to ≤64 mg/L, and thus, providing sufficient antimicrobial coverage against KPC-Kp. The CFRs of combination therapy were raised to > 80% in normal renal function and > 90% in renal impairment based on the PK/PD target of AUC_24_/MIC. Thus, empirical therapy in the treatment of infections caused by KPC-Kp with high MICs can use the combination regimens of FOS and TGC. Besides, drugs in combination could completely suppress all clones resistant to FOS at a low dose of 12 g/day [43]. Although the FOS daily dose of 18 g to 24 g in combination with TGC might be promising, these high doses may cause adverse side effects, such as hypokalemia and saline overload [44]. It is worth noting that it is still not fully elucidated if dose adjustment is needed for the CrCL of 40 to 80 ml/min. For patients with CrCL < 40 ml/min, a reduction of daily recommended dose is proposed [45]. As the means of simulated CrCL in our study was 40–55 ml/min for the decreased renal function cohort, these high doses in combination may be a safe and effective therapeutic method for management of difficult-to-treat infections in such patients.

TGC showed limited in vitro activity against KPC-Kp. The data of TGC MIC, used in the MCS, was relatively high compared with other studies [10, 46]. Thus, the recommended standard dosing regimen of TGC (100 mg loading dose followed by 50 mg q12h) failed to achieve PK/PD targets for KPC-Kp in combination therapy. Currently, the role of TGC in treating critically ill patients is still controversial [12]. In 2013, the Food and Drug Administration (FDA) reported an increased risk of death associated with TGC use [47]. The reason may be the suboptimal dosing regimens and relatively high MICs in certain bacterial strains [48, 49]. Yamashita. et al. indicated peak TGC serum levels were low (0.63–1.4 mg/L) after administrating the standard dosing regimen of TGC [50]. Thus, it is still essential to evaluate the efficacy of TGC dosing regimens owing to the above situation and limited treatment options. Consistent with previous studies, our findings indicated that standard TGC dosing regimen was suboptimal, while an increase of the daily dose could achieve better PTA and CFR [46]. High dose has been evaluated in the treatment of CRKP and found lower mortality and better clinical responses compared with the recommended standard dosage [51, 52]. Due to the long t_1/2_ (42 h following multiple doses) and linear PK characteristics of TGC, once daily high dose TGC regimens were also simulated in our study and reached favourable CFR in combination therapy. Thus, its clinical value as an option of last resort for treating multidrug-resistant isolates is worthy of exploration. In view of these results, high dose is essential to obtain maximum concentration-dependent killing, especially for Carbapenem-resistant organisms with an MIC of 2 mg/L. But the incidence of adverse events, mainly concerning gastrointestinal disorder, was elevated in the high TGC group [51]. Of note, the difference in serious adverse events was not statistically significant. TGC is well tolerated at high dose. Similar clinical outcomes of high-dose vs low-dose TGC were also described in a meta-analysis study, including 1041 patients [53]. It has been suggested that no dose adjustment was required for TGC in renal or hepatic impairment, unless there is severe hepatic dysfunction. From our simulated results, TGC 200 mg loading dose followed by 100 mg q12h in combination with FOS 8 g q8h in normal renal function or FOS 4 g q8h in renal impairment might be reasonable in empirically treating critically ill patients infected with KPC-Kp, so as to maximize a favorable clinical response and minimize exposure-related toxicity. In the future, well-designed studies especially randomized controlled trials (RCTs) are required to establish the effectiveness and safety of high-dose TGC.

The clinical breakpoint of CST at present is 2 mg/L for *Enterobacteriaceae*. However, in such situation, only the two loading regimens achieved PTA values higher than 90% in the renal impairment. This would be expected to increase the likelihood of acute renal failure. Presently, the daily dose is suggested to be reduced in patients with decreased renal function. Our findings showed that CMS dosing regimens in combination with FOS led to a CFR in the range of 60–80% and 80–92% for normal renal function and renal impairment, respectively. Similar clinical cure rate with no significant renal toxicity was observed in patients with sepsis due to Gram-negative bacteria susceptible only to CST and treated with 4.5 million IU q12h [54]. However, lower clinical cure rates (57–75%) have been reported in the low CMS dose (2.2–6 million IU/day) group [55, 56]. Such low daily doses always failed to produce sufficient drug exposures to reach the PK/PD target for isolates with an MIC of 0.5–1 mg/L in our study. It has been stated that the current recommended dose of CMS by manufacturers is associated with suboptimal concentrations in a large number of the patients [57]. Worsely, such sub therapeutic concentrations often resulted to the amplification of colistin-resistant subpopulations in heteroresistant strains [58]. Combination therapy is still needed in view of our findings and previous studies. Moreover, a previous meta-analysis indicated that mortality was significantly higher with polymyxin monotherapy compared with combination therapy with TGC, FOS or aminoglycosides, especially for *K. pneumoniae* bloodstream infection [59]. Considering that increasing use of CMS and the spread of *mcr-1* gene in plasmid might be leading to the emergence of CST resistance worldwide [60, 61], the combination with FOS can take into account the antibacterial efficacy and the reduced CMS daily dose so as to decrease the likelihood of the risk of nephrotoxicity, which is instructive in managing patients with decreased renal function.

There are several limitations to this study. First, the population PK model of FOS was developed form 12 enrolled patients with a total of 515 plasma samples [27]. And for TGC and CMS, 146 and 105 patients were included in their studies [29, 31]. Thus, the rich PK properties of FOS was not fully evaluated, meaning that other relevant covariates might not be included in the model. Second, the MICs of the KPC-Kp populations isolated from the three hospitals may not be representative of the MIC distributions in other regions. Third, a precise prediction of the efficacy of antibiotics against KPC-Kp is challenging because of the complicated condition in critically ill patients. Although the host immune response was not evaluated in our study, the presence of a competent immune system can markedly increase the efficacy of drugs against bacterial infections [62]. Moreover, combination therapy was often used for such patients in clinical practice. In addition, the PK/PD targets used in our study might be not fully elucidated, as the PK/PD targets used in our study were established for monotherapy. Further studies, including PK/PD simulations, animal models, and clinical trials, are urgently needed to evaluate the efficacy and toxicity of FOS, TGC and CMS against CRKP.

## Conclusion

To our knowledge, this is the first and largest study to assess the combined treatment of FOS with TGC or CMS against KPC-Kp using a bayesian-based dosing in China. Loading dose is essential for TGC and CMS, and high dose TGC (200/400 mg loading dose followed by 100 mg q12h/200 mg q24h) and CMS (9 million IU loading dose followed by 4.5/3 million IU q12h) in combination with FOS is needed to provide sufficient antimicrobial coverage against KPC-Kp.

## Supplementary Information

**Additional file 1: **Supplementary material associated with this article can be found in **Table S1.** and **Figure S1.**

**Additional file 2.**

## Data Availability

The datasets used and analysed during the current study are available from the corresponding author on reasonable request.
