# Supplementary material for: Optimal empiric treatment for KPC-2-producing Klebsiella pneumoniae infections in critically ill patients with normal or decreased renal function using Monte Carlo simulation
Source: BMC Infect Dis. 2021 Mar 26;21:307. doi: 10.1186/s12879-021-06000-2 (PMC8004468; doi:10.1186/s12879-021-06000-2)
Supplement: Supplementary file 1 — Additional file 1: Supplementary material associated with this article can be found in Table S1. and Figure S1. [file 12879_2021_6000_MOESM1_ESM.zip › Figure S1.pdf]

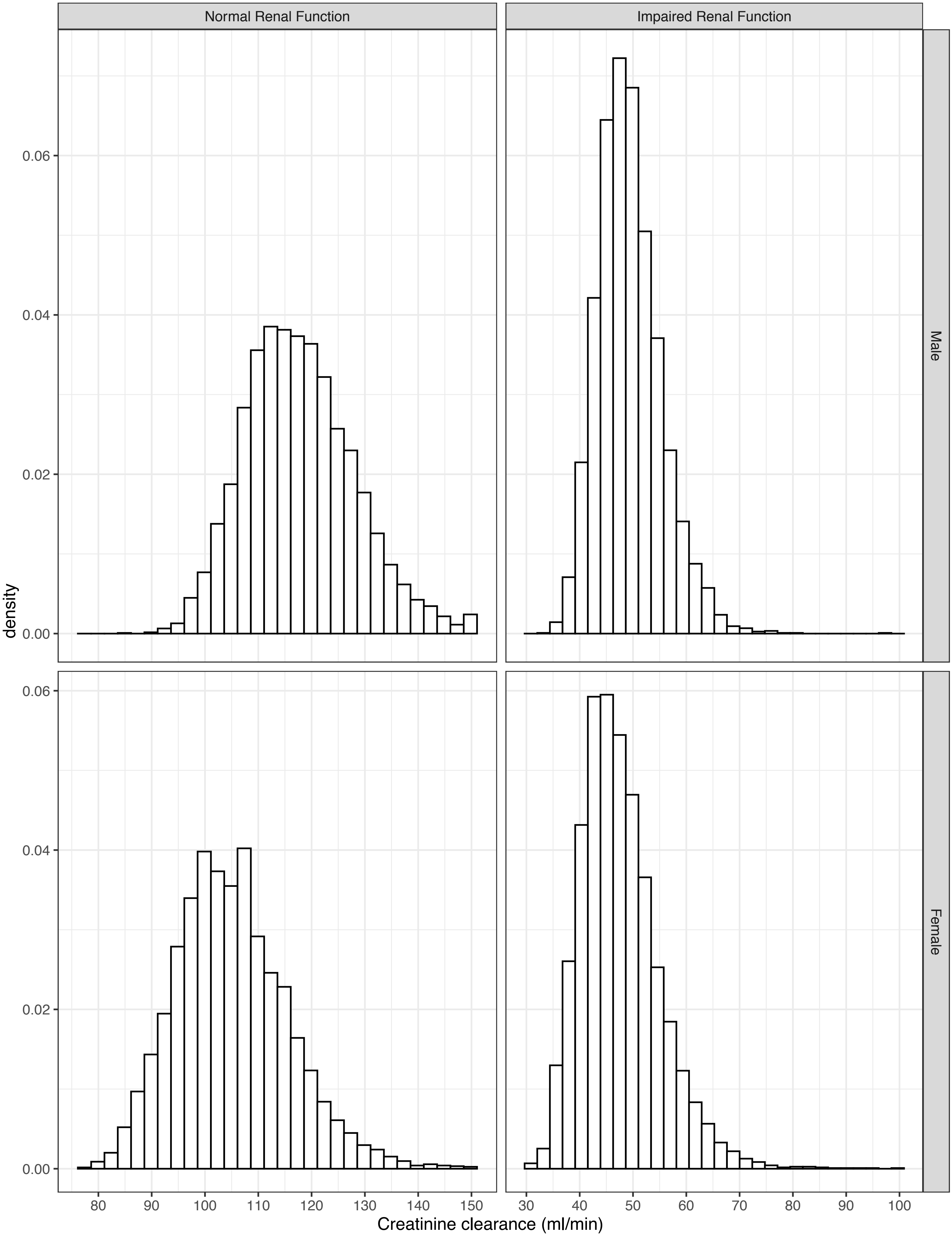

Figure S1. The distribution of creatinine clearance calculated by the modification of renal disease equation in critically ill elderly male (top) and female (bottom) patients with normal renal function (left) and renal impairment (right).
