# Supplementary material for: Optimal empiric treatment for KPC-2-producing Klebsiella pneumoniae infections in critically ill patients with normal or decreased renal function using Monte Carlo simulation
Source: BMC Infect Dis. 2021 Mar 26;21:307. doi: 10.1186/s12879-021-06000-2 (PMC8004468; doi:10.1186/s12879-021-06000-2)
Supplement: Supplementary file 1 — Additional file 1: Supplementary material associated with this article can be found in Table S1. and Figure S1. [file 12879_2021_6000_MOESM1_ESM.zip › Supplementary Table.docx]

| **Table S1.** MICs of KPC-2-producing *K. pneumoniae* against tested agents, mono drugs or combination drugs. | | | | |
| --- | --- | --- | --- | --- |
| Antimicrobial agent | Antimicrobial mono and combination drugs | MIC_50_ | MIC_90_ | MIC range |
| FOS | monodrug | 256 | 1024 | 1-4096 |
|  | FOS+TGC | 16 | 64 | ≤0.06-2048 |
|  | FOS+CST | 32 | 256 | ≤0.06-1024 |
| TGC | monodrug | 2 | 4 | 0.5-16 |
|  | TGC+FOS | 1 | 4 | ≤0.06-4 |
| CST | monodrug | 0.25 | 0.5 | 0.125-32 |
|  | CST+FOS | 0.25 | 0.5 | ≤0.06-1 |

FOS, fosfomycin; TGC, tigecycline; CST, colistin.
