# Supplementary material for: Optimal empiric treatment for KPC-2-producing Klebsiella pneumoniae infections in critically ill patients with normal or decreased renal function using Monte Carlo simulation
Source: BMC Infect Dis. 2021 Mar 26;21:307. doi: 10.1186/s12879-021-06000-2 (PMC8004468; doi:10.1186/s12879-021-06000-2)
Supplement: Supplementary file 2 — Additional file 2. [file 12879_2021_6000_MOESM2_ESM.pdf]

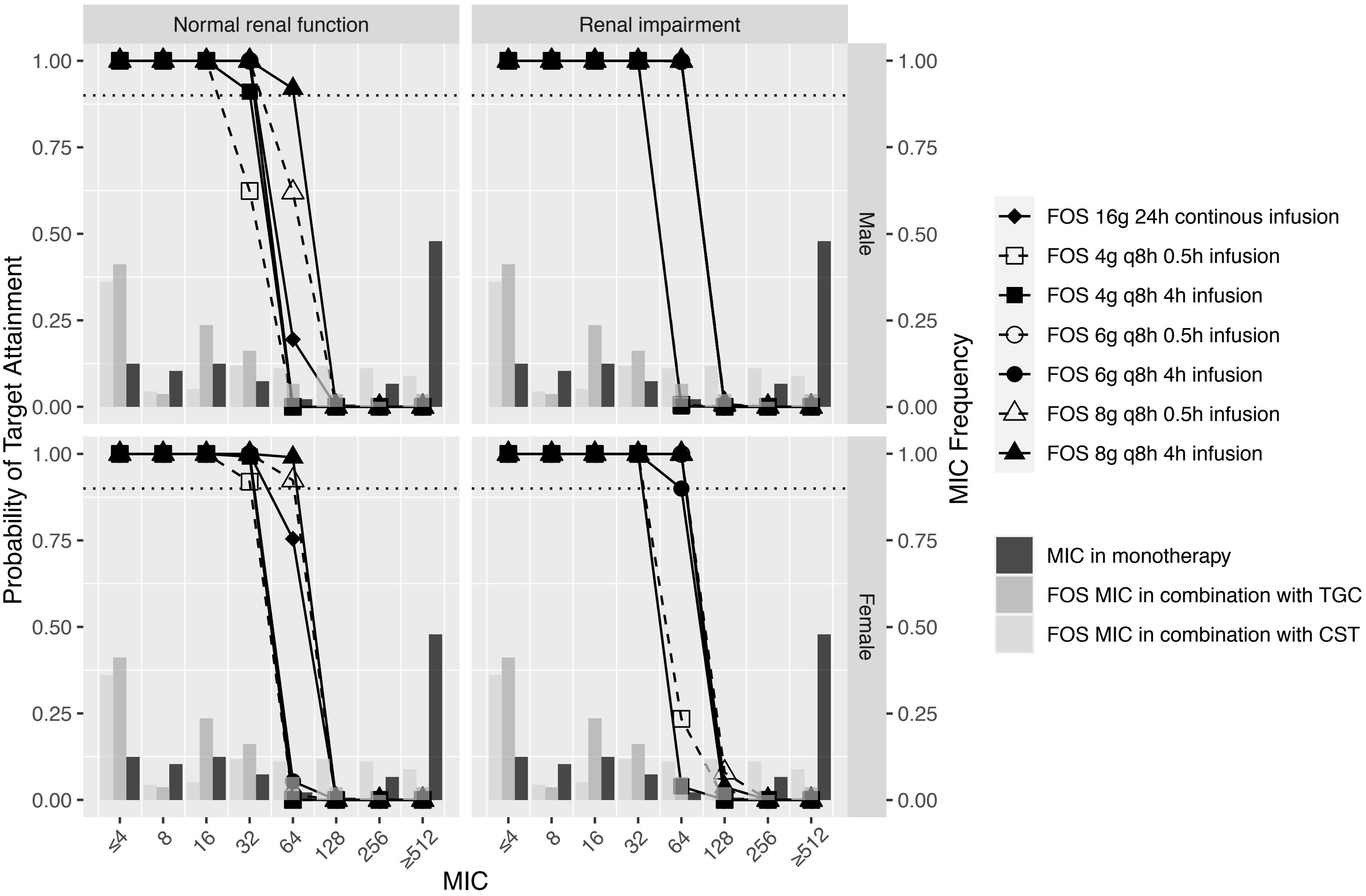

Figure S2. The MIC distribution of FOS in monotherapy or combination with TGC or CMS against 136 KPC-producing *Klebsiella pneumoniae,* and probability of target attainment (PTA) of 70% fT>MIC for FOS dosing regimens in critically ill elderly patients with normal renal function (Left) and renal impairment (right). Open symbols indicate a 0.5-h infusion, and filled symbols indicate a prolonged infusion.The dotted line indicates the PTA of 0.9. FOS, fosfomycin; TGC, tigecycline; CST, colistin.
